# Do Humans and Deep Convolutional Neural Networks Use Visual Information Similarly for the Categorization of Natural Scenes?

**DOI:** 10.1111/cogs.13009

**Published:** 2021-06-25

**Authors:** Andrea De Cesarei, Shari Cavicchi, Giampaolo Cristadoro, Marco Lippi

**Affiliations:** ^1^ Department of Psychology University of Bologna; ^2^ Department of Mathematics and Applications University of Milano‐Bicocca; ^3^ Department of Sciences and Methods for Engineering University of Modena and Reggio Emilia

**Keywords:** Visual categorization, Convolutional neural networks, Spatial frequencies

## Abstract

The investigation of visual categorization has recently been aided by the introduction of deep convolutional neural networks (CNNs), which achieve unprecedented accuracy in picture classification after extensive training. Even if the architecture of CNNs is inspired by the organization of the visual brain, the similarity between CNN and human visual processing remains unclear. Here, we investigated this issue by engaging humans and CNNs in a two‐class visual categorization task. To this end, pictures containing animals or vehicles were modified to contain only low/high spatial frequency (HSF) information, or were scrambled in the phase of the spatial frequency spectrum. For all types of degradation, accuracy increased as degradation was reduced for both humans and CNNs; however, the thresholds for accurate categorization varied between humans and CNNs. More remarkable differences were observed for HSF information compared to the other two types of degradation, both in terms of overall accuracy and image‐level agreement between humans and CNNs. The difficulty with which the CNNs were shown to categorize high‐passed natural scenes was reduced by picture whitening, a procedure which is inspired by how visual systems process natural images. The results are discussed concerning the adaptation to regularities in the visual environment (scene statistics); if the visual characteristics of the environment are not learned by CNNs, their visual categorization may depend only on a subset of the visual information on which humans rely, for example, on low spatial frequency information.

## 1 Introduction

Making sense of the world and taking appropriate decisions is essential for adaptive behavior and survival. Concerning vision, this means making sense of the light and shade which is projected onto the retina. Despite overwhelming visual variety (due to viewpoint, illumination, and so on), in most cases, humans and other animals can make sense of the outer world. Moreover, vision is acquired early in phylogenetic development (Gehring, 2014), and visual understanding is learned early in the development of individuals (Bernstein, Loftus, & Meltzoff, 2005). How visual understanding is achieved is the object of study in diverse disciplines, such as general psychology, visual neuroscience, and computer science. Recently, these disciplines have shown a converging interest in artificial simulations of vision, namely deep convolutional neural networks (CNNs), which compete with humans in terms of capability to classify visual scenes (e.g., He, Zhang, Ren, & Sun, 2016). However, a similarly accurate performance can depend on different underlying processes, and an active line of studies investigates the degree of similarity between human and artificial vision (Dodge & Karam, 2019; Geirhos et al., 2018; Rajalingham et al., 2018; Tadros, Cullen, Greene, & Cooper, 2019). Here, we address the functional similarity between CNN and human vision, and, more specifically, investigate whether humans and CNNs use the same visual information for the categorization of natural scenes.

Categorizing natural scenes consists of classifying real‐world views into separate predefined classes. A vast body of literature has examined the types of categorization that can be carried out, for example, distinguishing basic, superordinate, and subordinate levels of categorization (Rosch, Mervis, Gray, Johnson, & Boyes‐Braem, 1976). Previous studies observed that superordinate categorization (e.g., distinguishing “animals” from “vehicles”) can be performed efficiently with briefly presented stimuli (De Cesarei, Codispoti, Schupp, & Stegagno, 2006; Rousselet, Macé, Thorpe, & Fabre‐Thorpe, 2007; Thorpe, Fize, & Marlot, 1996; VanRullen & Thorpe, 2001), engages high‐order visual areas (Codispoti, Ferrari, Junghöfer, & Schupp, 2006; De Cesarei, Cavicchi, Micucci, & Codispoti, 2019; Fize et al., 2000), and has electrocortical correlates that include both an earlier component which is related to the processing of stimulus‐driven scene statistics, and a later component which is independent from the bottom‐up differences and reflects a more task‐related semantic analysis (De Cesarei et al., 2019; Rousselet et al., 2007; VanRullen & Thorpe, 2001).

**Fig. 1:**
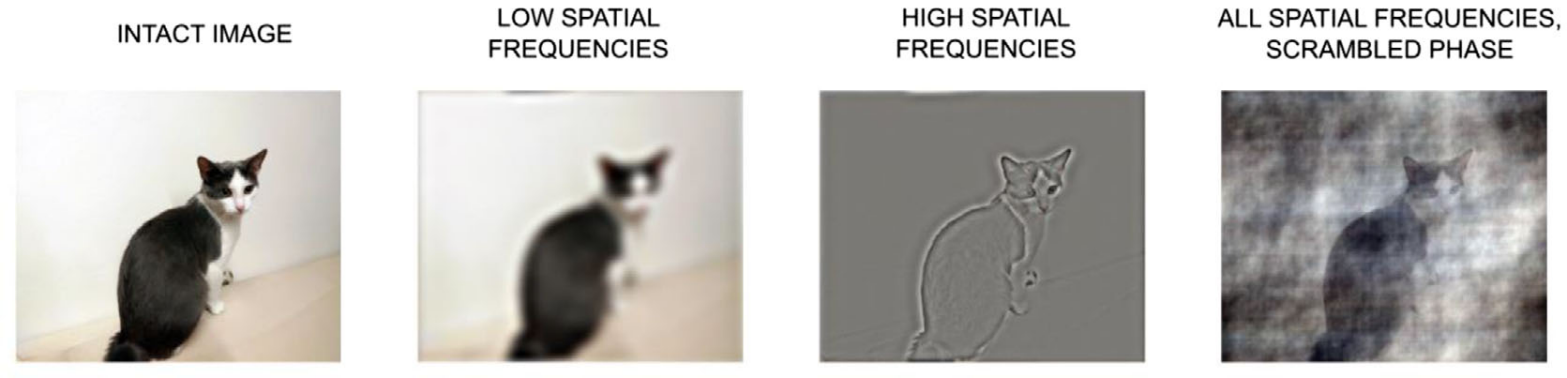
Example of an intact picture, and of degraded versions of the same picture. In this example, low‐pass filtering, high‐pass filtering, and phase scrambling are applied by transforming pictures from the image space (how they appear) to the frequency space (how they are composed) using Fourier transforms. If the spatial frequency space is altered, either by filtering some frequencies (e.g., low or high) or by manipulating the phase, and then the picture is transformed back from the frequency to the image space, a degraded version of the original picture is obtained

Visual information is represented on several spatial scales, ranging from gross silhouettes to fine details, and these scales can be exploited for the task at hand; some tasks may rely on coarse shapes, while others may require the analysis of fine details (De Cesarei & Loftus, 2011; De Cesarei et al., 2019; Navon, 1977; Schyns & Oliva, 1994). Spatial scales are best described by spatial frequencies (Field, 1987; Shulman & Wilson, 1987), which are one of the organizing factors in the columnar architecture of the visual cortex (Blakemore & Campbell, 1969). Spatial frequencies measure the relationship between the visual area which is occupied by an element compared to a reference area (e.g., the whole scene), and can be used to select a specific scale of visual information (e.g., high spatial frequencies [HSFs] for tiny details). The visual system can understand the meaning of a scene based on several spatial frequencies. In the example in Figure 1, we can understand that there is a cat both when we focus on the dark silhouette against the wall (low spatial frequencies, LSFs; e.g., Sanocki, 1993), and when we focus on the finer contours of the animal (HSFs; Berman, Golomb, & Walther, 2017). A vast amount of literature has examined the relative use over time of low and high spatial frequencies, showing that global (operationalized as LSF) elements of a scene are processed before, and interfere with the processing of, local (operationalized as HSF) elements of a scene (De Cesarei & Loftus, 2011; Kimchi, 1992; Navon, 1977; Sanocki, 1993; Schyns & Oliva, 1994). Finally, while the amplitude of spatial frequencies measures the presence of sized elements in the visual input, the composition of an image is also described by its spatial frequency phase, which provides information as to the relative position of sized elements within the visual input and is related to the presence of higher‐order regularities in the appearance of a visual stimulus (Field, 1987). While categorization performance is relatively robust to changes in spatial frequency amplitude, disruption of phase leads to an increase in computational load and in a severe dampening of image understanding (Arsenault, Yoonessi, & Baker, 2011; Codispoti, Micucci, & De Cesarei, 2021; De Cesarei, Peverato, Mastria, & Codispoti, 2015; Joubert, Rousselet, Fabre‐Thorpe, & Fize, 2009; VanRullen, 2011).

Recently, deep neural networks (DNNs; Hinton & Salakhutdinov, 2006; Krizhevsky, Sutskever, & Hinton, 2012; LeCun, Bottou, Bengio, & Haffner, 1998; Rumelhart, Hinton, & Williams, 1986; Zhao, Zheng, Xu, & Wu, 2019) have gained popularity among artificial models of vision, partially due to their extremely high accuracy in visual tasks. DNNs acquire information from an input layer and generate a decision (e.g., an animal/vehicle prediction value) from an output layer; moreover, several layers exist between the input and output layers. In deep CNNs, these layers constrain the massive input information through progressively more restricted layers, until a single output is generated. Each layer contains units which are connected to each other according to certain weights, which are adjusted through learning. In turn, several training schedules exist, for example, unsupervised, reinforcement‐based, semi‐supervised, or supervised. The training phase is meant to allow the network to learn those regularities which are present in the training dataset, such as natural scenes; further learning can be achieved later, for example, by fine‐tuning the network to a more specialized task. When the network is tested with new stimuli, the accuracy of the CNN is often close to that of humans (e.g., Krizhevsky et al., 2012). Studies which directly compared visual representations in humans and CNNs indicated a similarity between the hierarchical analysis of visual information in hidden CNN layers and the sequence of stages of visual processing in the human brain (Cadieu et al., 2014; Cichy, Khosla, Pantazis, Torralba, & Oliva, 2016; Groen et al., 2018; Güçlü & van Gerven, 2015; Khaligh‐Razavi & Kriegeskorte, 2014; Yamins & Di Carlo, 2016; Yamins, Hong, Cadieu, & DiCarlo, 2013). These similarities suggest that CNNs are useful tools not only for solving practical engineering tasks, but also for modeling biological vision (VanRullen, 2017; Wardle & Baker, 2020). However, this brings in the issue of the comparison between CNN and human visual processing, for both theoretical and applied reasons. On the applied side, an artificial system which can replicate both the successes and errors of humans might be used to examine the likelihood of accurate perception when visual conditions are degraded, for example, in the fog or dust, in peripheral vision, or in low‐contrast conditions. On the theoretical side, the modeling of mental operations by artificial algorithms has long been used as a tool for understanding human cognition, eliminating “hidden elements of the anthropomorphically subjective” (Hull, 1943, p. 27) and allowing for “simulation of behavior” (e.g., Abelson & Carroll, 1965), and more recently for computational accounts of visual processing (e.g., Deco & Rolls, 2002). In this line, CNNs proved themselves to be useful models for computational simulation of vision, both in terms of cortical activity (e.g., Wardle & Baker, 2020) and understanding the representation of stimulus dimensions (e.g., Kubilius, Bracci, & Op de Beeck, 2016).

However, differences in visual processing have also been observed between humans and CNNs. For instance, CNNs can be “fooled” to categorize meaningless textures as real objects with high confidence (Nguyen, Yosinski, & Clune, 2015). Moreover, adding visual noise which does not impact human categorization prevented CNNs from correctly recognizing intact images (Szegedy et al., 2014). More specifically, in these studies, “adversarial images” are presented to CNNs, and these images contain noise which is imperceptible to humans, but alters the CNN visual representation enough to lead it to an incorrect classification. However, these previous studies lacked a quantitative comparison between humans and CNNs. Recently, some studies have directly compared visual processing in humans and CNNs, focusing on the final performance achieved by the classificator rather than on the internal (brain or layers) visual input representation that is achieved (Dodge & Karam, 2019; Geirhos et al., 2018; Kubilius, Bracci, & Op de Beeck, 2016; Rajalingham et al., 2018; Tadros et al., 2019). When directly comparing humans and CNNs, Dodge and Karam (2019) and Tadros et al. (2019) observed that while CNN visual classification of intact stimuli is on a par with that of human participants, it drops significantly with degraded pictures, and that CNN errors have little correlation with human ones. A similar result was observed by Geirhos et al. (2018), who additionally observed that, while retraining the CNN on the distortion type it would be tested on ameliorated its performance, this improvement did not generalize to other distortion types. Moreover, Rajalingham and colleagues (2018) observed low image‐level agreement between humans and CNNs. Altogether, these previous results suggest that functional similarities between human and CNN vision may be restricted to some types of visual information.

### 1.1 The research problem

Here, we examine the use of visual information by humans and CNNs, focusing specifically on the amplitude and phase of the image spatial frequency spectrum. The task, for a sample of human participants and a total of four CNNs, was to categorize pictures as representing animals or vehicles (superordinate categorization). We chose two categories (animals and vehicles), which were used in the previous literature (De Cesarei et al., 2019; Thorpe et al., 1996; VanRullen & Thorpe, 2001), and are familiar to human participants. Pictures could be intact or degraded through low‐pass filtering, high‐pass filtering, or phase‐scrambling. High‐ and low‐pass filters remove global shapes and sharp details, respectively, and were used here to examine the role of spatial scales (ranging continuously from high to low) in performance accuracy. Moreover, several studies indicate that not only the amplitude, but also the phase of the spatial frequency spectrum is important for picture understanding (Codispoti et al., 2021; De Cesarei et al., 2015; Joubert et al., 2009). Specifically, if the phase of an image is altered, its spatial frequency components are displaced even if their amplitude is not altered; surprisingly, no study exists, to our knowledge, that has manipulated the phase of the spatial frequency spectrum while assessing categorization by CNNs. In all conditions (amplitude filtering; phase scrambling), we reasoned that if the sources of information for accurate visual categorization in humans and CNNs are similar, then comparable dampening of performance should be observed as visual information is similarly degraded.

## 2 General method

### 2.1 Task

In all experiments, humans and CNNs categorized pictures as containing animals or vehicles. Scene understanding was dampened by degrading the picture through high‐pass filtering (HSF), low‐pass filtering (LSF), or phase scrambling.

### 2.2 Pictures

#### 2.2.1 Test stimuli

A total of 576 pictures were selected from the Internet to test the categorization of natural scenes by humans and CNNs. Pictures portrayed one or more foreground objects in real‐world environments. In half of the pictures, the foreground objects were animals, whereas in the other half, they were vehicles. Pictures were in color and were balanced for brightness and contrast (pixel intensity *M* = 128, *SD* = 72.11). Each picture was resized to 431×323 pixels before being degraded.

#### 2.2.2 Fine‐tuning of CNNs

For the fine‐tuning phase of the CNNs, a set of 3130 color pictures was obtained from the two synsets Animals (1571 images) and Vehicles (1559 images) in the ImageNet database (Deng et al., 2009). These pictures were also resized to 431×323 pixels, and balanced for brightness and contrast as for test pictures.

#### 2.2.3 Picture degradation

Different versions of each picture were created by manipulating the amplitude or the phase of the spatial frequency spectrum. Spatial frequency filtering was carried out using Matlab routines (De Cesarei, Mastria, & Codispoti, 2013; Loftus & Harley, 2005), and different versions of the same picture were created by applying a low‐ or high‐pass spatial frequency filter, varying in filter threshold. These filters pass all spatial frequencies up to a frequency value of F0, when spectral amplitude begins to be attenuated with a parabolic falloff. Spectral amplitude is completely filtered out when an F1 level is reached, and this happens three octaves above or below the F0 parameter in the case of LSF and HSF filters, respectively. Hereafter, we report F1, which is the value at which spectral amplitude is totally filtered out. For human participants, four degradation levels were created for each filtering condition, and the filtering thresholds were decided based on the previous research (De Cesarei & Codispoti, 2011; De Cesarei & Loftus, 2011) and on pilot data on a separate sample of participants, with the aim of identifying values that sampled the rising part of the psychometric function. For the HSF condition, F1 values were 120.68, 85.33, 60.34, and 10.67 cpi. For the LSF condition, F1 values were 5.66, 9.19, 14.93, and 64 cpi. For the phase scrambling condition, we used a weighted mean phase algorithm (Dakin, Hess, Ledgeway, & Achtman, 2002). Specifically, the phase spectrum of the original image was combined with the phase of random noise, and the relative weight of the original and the random phase ranged from 0% (original picture) to 100% (only noise). Then, the weighted phase and the original amplitude spectrum were recombined to obtain the final phase‐scrambled image that was used in the experiments. The phase weighting parameters were 70%, 65%, 60%, and 40% for human participants. Each degraded picture was then adjusted in brightness and contrast, in order to span the entire 0–255 brightness range.

For CNNs, 15 degradation levels were used, which were chosen to cover the full accuracy degradation function, four of which matched the levels seen by human participants. Therefore, for the HSF condition, we used 170.67, 120.68, 85.33, 60.34, 42.67, 30.17, 21.33, 15.09, 10.67, 7.54, 5.33, 3.77, 2.67, 1.89, and 1.33 cpi as F1 values. For the LSF condition, we used 2.14, 3.48, 5.66, 9.19, 14.93, 24.25, 39.40, 64.00, 103.97, 168.90, 274.37, 445.72, 724.08, 1176.27, and 1910.85 cpi as F1 values. Phase weighting parameters were 85%, 80%, 75%, 70%, 65%, 60%, 55%, 50%, 45%, 40%, 35%, 30%, 25%, 20%, and 15%. With these degradation parameters, we had four conditions in which human and artificial degradation parameters matched, and were therefore comparable (for HSF: 120.68, 85.33, 60.34, and 10.67 cpi; for LSF: 5.66, 9.19, 14.93, and 64 cpi; for phase scrambling: 70%, 65%, 60%, and 40%). For comparison with previous studies, the filter thresholds in cycles per degree in the human experiment were 10.40, 7.36, 5.20, and 0.92 cpd (for high‐pass filter) and 0.49, 0.79, 1.29, and 5.52 cpd (for low‐pass filter).

### 2.3 Human participants and artificial networks

#### 2.3.1 Human participants

A total of 14 participants (six females) took part in the human experiment (age *M* = 26.21, *SD* = 3.28). All participants had normal or corrected‐to‐normal vision, and none of them reported neurological or psychopathological problems. All participants provided written informed consent. The experimental protocol conforms to the declaration of Helsinki and was approved by the Bioethical Committee of the University of Bologna.

##### 2.3.1.1 Stimuli and apparatus

Stimuli were selected and degraded as indicated in the General method section. Moreover, a total of nine additional pictures were selected and degraded for an initial practice phase, to allow participants to familiarize themselves with the filters used in the study. The experiment was programmed using OpenSesame (Mathôt, Schreij, & Theeuwes, 2012), and presented on an 11.6” monitor with a 1280×720 resolution, placed 50 cm away from the participant, yielding a visual angle of 14.8° (horizontal) x 11.6° (vertical).

##### 2.3.1.2 Procedure

The experiment consisted of a single block of 576 trials. Each trial began with the presentation of a picture that remained on the screen for 800 ms. After picture offset, a question appeared, asking participants whether the picture they observed portrayed an animal or a vehicle; this question remained on the screen until the participant responded. Participants could respond by pressing one of two alternative keys (n, for the “animal” response or m, for the “vehicle” response) on a computer keyboard. The instructions encouraged participants to give accurate rather than fast responses. No feedback was given regarding the accuracy of the response. Subsequently, participants had to rate their confidence in their response, using a scale from 1 (not confident at all) to 5 (absolutely confident). Confidence data closely replicated the pattern which was observed for accuracy, and are not reported here. After 500 ms of blank screen, the following trial began.

Each participant saw each picture only once, in only one degradation condition. Across participants, each picture was seen in each combination of type and level of degradation. The order of pictures was pseudorandomized, to avoid repetition of the same category of pictures for more than six consecutive trials. Each combination of type and level of degradation (e.g., highest level of phase scrambling) was presented for a maximum of five consecutive trials.

#### 2.3.2 Artificial networks

Here, we used CNNs which are widely used in the computer vision and neuroscience literature: AlexNet, VGG‐16, ResNet‐50, and DenseNet‐201. For each of these nets, we used specific implementations that had been pretrained using the ImageNet database, a very large (more than 14 million images) set of semantically labeled pictures. Each CNN is described in the following paragraphs. For all the considered architectures, we adopted the following experimental setup, which is customary in computer vision applications that exploit pretrained architectures (Oquab et al., 2014). We removed the top fully connected layer of each network, plugging a new fully connected layer with 128 neurons on top, and adding a final layer with a single neuron, as the aim was to perform binary classification. Between the last two layers, a dropout equal to 0.5 was applied, as it has become customary to improve the generalization capabilities of the networks (Srivastava, Hinton, Krizhevsky, Sutskever, & Salakhutdinov, 2014). For each training example given in input, the backpropagation algorithm (Rumelhart et al., 1986) compares the target of the prediction with the value computed by the network, and it back‐propagates the information from the output nodes to the input nodes of the network by adjusting the weights, so as to move the predicted output closer to the desired value. We first trained the newly introduced layer only, for three epochs, with the Adam optimizer (Kingma & Ba, 2017) with an initial learning rate equal to 0.00005. Then, we fine‐tuned the whole architecture for an additional three epochs, with a slightly larger learning rate, namely 0.0001. Only for AlexNet, a few more epochs were necessary (up to 15) until the learning curve stopped improving. For all the networks except for AlexNet, we used the Keras implementation with Tensorflow as backend (version 2.4.3 for Keras, version 2.2.0 for Tensorflow). For AlexNet, which is a more dated model, and is not present in the Keras suite, we used an external library (https://github.com/heuritech/convnets‐keras) that works with Theano (version 1.0.4) as backend. The code used in the experiments is freely available for research purposes at the following repository: https://github.com/marcolippi83/cnns‐animals‐vehicles.

##### 2.3.2.1 AlexNet

The first CNN tested was AlexNet (Krizhevsky et al., 2012), which was introduced in 2012 in the context of the ImageNet Large Scale Visual Recognition Challenge (ILSVRC), yielding a remarkably high‐level performance. This net contains a total of eight layers, comprising layers doing convolution and max pooling, and fully connected layers, and ending with a final softmax layer. This net has a very large number of parameters (about 61M).

##### 2.3.2.2 VGG‐16

The second CNN we used is VGG‐16 (Zhang, Zou, He, & Sun, 2015). This CNN has a deeper structure compared with AlexNet (16 vs. 8 layers); these layers contain two initial convolutional layers, which are followed by a single max pooling layer, and then a sequence of fully connected layers, until a final SoftMax layer outputs the classification. This net has a larger number of parameters compared with AlexNet (138M).

##### 2.3.2.3 ResNet‐50

The third CNN we used is ResNet, a deep architecture that is widely employed in computer vision applications (He et al., 2016). ResNet50 contains 49 convolutional layers and one fully connected layer. Moreover, residual networks, such as ResNet‐50, contain shortcut connections that skip one or more layers. These shortcut connections allow the network to be trained based on the value of residuals; the core idea of this architecture is that optimizing the residual mapping is easier than optimizing the original. Specifically, if the desired mapping for a layer is H(x), a different mapping F(x) = H(x) − x is employed, while later the input is summed back to F(x) to obtain H(x). Compared with VGG‐16, this net has a smaller number of parameters (25.5M).

##### 2.3.2.4 DenseNet‐201

Finally, we examined the performance of DenseNet (Huang, Liu, Maaten, & Weinberger, 2017), which is also widely employed in computer vision applications, and characterized by the presence of long‐range feedforward connections. In DenseNet, shortcut connections link each layer to all the other upper layers in the neural architecture. The architecture of DenseNet allows for an accurate classification with a relatively small number of parameters (20M).

#### 2.3.3 CNN fine‐tuning and test

First, we proceeded to fine‐tune the networks for the task at hand. Pretrained models can be further fine‐tuned for any other classification task, with the advantage of exploiting the weights of the lower layers, that have been learned on very large image collections (“transfer learning”; Oquab et al., 2014). Lower‐level and mid‐level layers in deep visual architectures, in fact, typically capture features that are independent of the task, such as edges, corners, or simple shapes.

Each CNN was fine‐tuned on a set of 3130 images, obtained from the two synsets Animals and Vehicles in the ImageNet database. To prevent overfitting, we performed early stopping by using 10% of the images for the validation set. Moreover, 10 slightly different versions of the same picture were used in order to increase the number of images in the fine‐tuning phase; this is a procedure which is often used to increase the learning that can be achieved by a CNN, without repeating exactly the same stimuli (Perez & Wang, 2017; Shorten, & Khoshgoftaar, 2019). To this end, we cropped 80% of each picture, beginning from the top left corner and moving the cropping window by 10% (vertically) and 6–10% (horizontally). Pictures that were used in the fine‐tuning phase were not used in the test phase. For each CNN, we performed 10 different fine‐tuning sessions with different random weight initializations for the newly introduced layers.

For the categorization test, each CNN was tested with the same pictures that were used for human participants. Therefore, each network was tested with 576 (pictures) × 3 (degradation) × 15 (levels) visual stimuli. For the analysis, we took the average of the categorization results for the 10 CNNs with randomized fine‐tuning weights as a group measure of CNN categorization accuracy. Each CNN test produced a total of 25,920 predictions, each one with a value ranging from 0 (label predicted vehicle) to 1 (label predicted animal). These values were rounded to the nearest integer (zero or one), in order to obtain a binary decision, which was used to compute accuracy and agreement with human categorization.

### 2.4 Data analysis

The aim of the data analysis was to describe the psychometric properties of the degradation‐accuracy functions, and to compute the agreement between humans and CNNs, in a categorization task. First, all data were fitted by a Weibull function, and the threshold of each function (80% accuracy) was computed. Confidence intervals (95%) for these parameters were obtained through a bootstrapping procedure, which resampled the estimated Weibull function 2000 times, each time combining original responses with flip coin (accurate/not accurate) results (Wichmann & Hill, 2001). Based on these bootstrapped distributions, 95% CIs were computed.

Then, we proceeded to compare the relative accuracy between artificial and human performance, and to analyze the level of agreement (or confusion) at the image‐level between humans and CNNs. In order to compare accuracy, we followed the approach suggested by Tadros et al. (2019). First, human and CNN accuracy data were aggregated per type and level of degradation. Then, for each of the reference cutoff levels to which both humans and CNNs contributed, the accuracy ratio was calculated as the ratio between artificial and human accuracy.

$$Accuracy\;Ratio\;=\left(\frac{k_{c}}{n_{c}}\right)\;/\;\left(\frac{k_{h}}{n_{h}}\right)$$

where *k* indicates the number of accurate trials and *n* is the number of total trials, for artificial classifiers (*c*) and human participants (*h*). A value of 1 indicates equal accuracy by humans and CNNs, while a value larger than 1 indicates better accuracy for CNNs, and a value smaller than 1 indicates a better performance for humans. Following Tadros et al. (2019), and using the log method described in Katz, Baptista, Azen, and Pike (1978) and Koopman (1984), we calculated standard deviation and *p*‐values, corrected (Bonferroni method) for four multiple comparisons, corresponding to the four degradation levels, which were used for each degradation type.

Additionally, we calculated the agreement in the image‐level responses between humans and CNNs. This analysis asks whether each image was categorized similarly by humans and CNNs, and outputs a value indicating the degree of agreement or disagreement. Clearly, if both systems have a close to full accuracy, high agreement will follow as a consequence (Tadros et al., 2019). Therefore, we calculated agreement as the relationship between observed (image‐level match) and expected (relationship between accuracies) agreement. Agreement calculation was done in three steps. First, for each human participant, type, and level of manipulation, we computed the *observed agreement* between the participant's decision for each individual picture and the decision of the CNN regarding the same picture. Then, we computed the *expected agreement* between humans and CNNs, following Tadros et al. (2019).

$$Expected\;Agreement=n_{h}\;\left(\left(\frac{k_{c}}{n_{c}}\right)\left(\frac{k_{h}}{n_{h}}\right)+\left(\frac{w_{c}}{n_{c}}\right)\left(\frac{w_{h}}{n_{h}}\right)\right)$$

where *k*, *w*, and *n* stand for correct responses, wrong responses, and total number of trials, respectively, and *h* and *c* stand for human and artificial. With this approach, expected agreement is calculated as the product of the correct human and artificial responses plus the product of incorrect artificial and human responses, and thus depends on the respective accuracy level of the two classificators (human and artificial). In a third and final step, observed and expected agreement are compared as a ratio, using the same approach as that used for accuracy (Tadros et al., 2019). Ratio values close to 1 indicate chance agreement between humans and CNNs. Values lower than 1 indicate that observed image‐level agreement is lower than expected based on accuracy, for example, in the case of systematic disagreement, while values higher than 1 are expected if the two classificators (human and artificial) agree on image categorization more than expected based on categorization accuracy.

## 3 Results

**Fig. 2:**
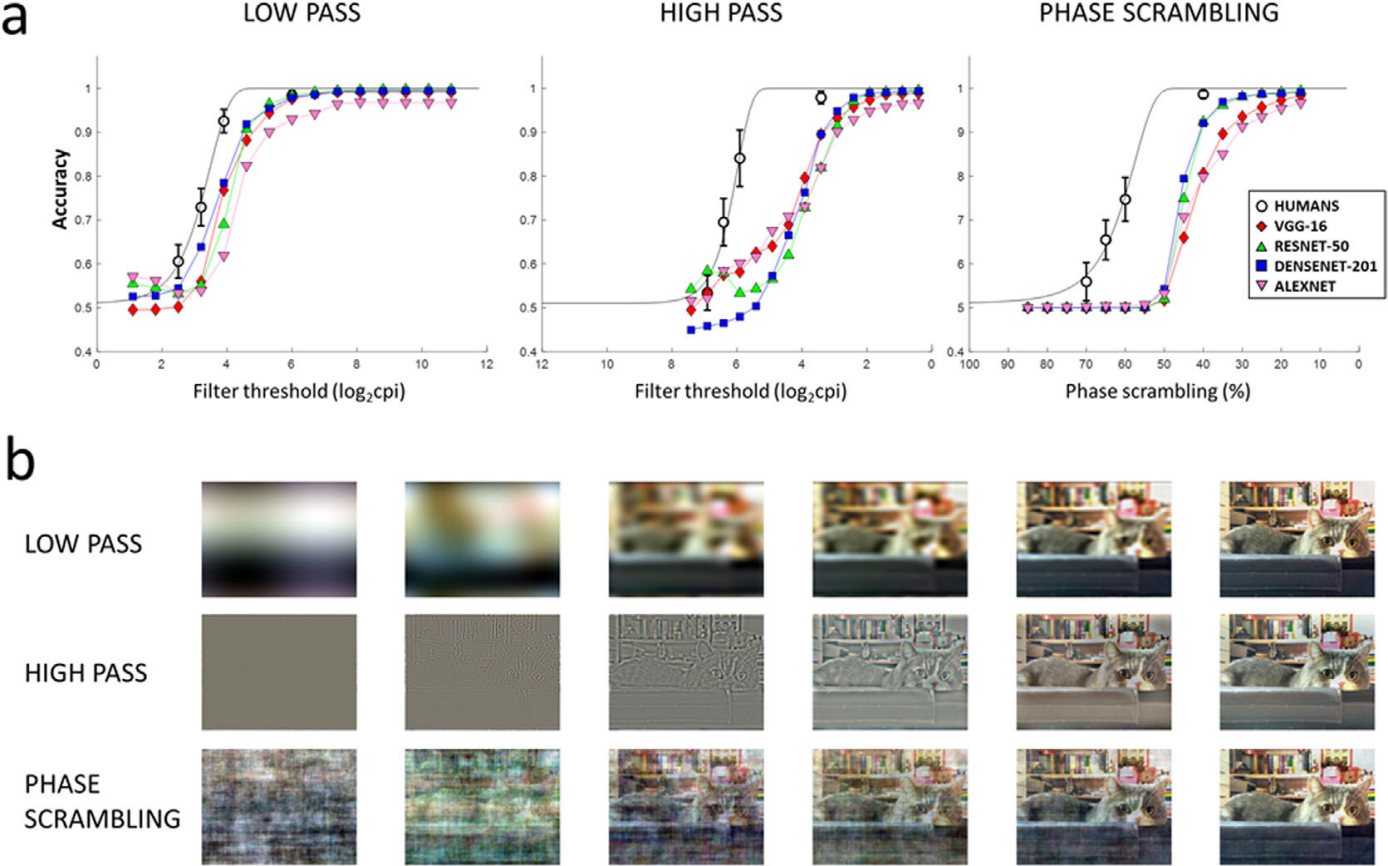
a) Degradation‐accuracy function for each CNN. The straight black line represents the Weibull fit to the human data. Error bars for human data represent confidence intervals. (b) Examples of the degraded images for six levels which are representative of the degradation range, in each of the degraded conditions.

Results for humans and CNNs are reported in Figure 2. Both human and CNN data followed Weibull psychometric functions, the threshold parameters of which are reported in Table 1. For low‐passed pictures, all CNNs showed a psychometric function, which was shifted toward higher low‐pass cutoffs compared with human participants, indicating that less degraded pictures were needed in order to achieve a similarly good categorization performance. Corroborating this visual impression, the threshold parameter for all CNNs was outside 95% of human CI. Concerning high‐passed pictures and phase‐scrambled ones, threshold parameters deviated even more substantially from human data.

**Table 1:** Psychometric threshold for the degradation‐accuracy function for normal pictures

	Low pass			High pass			Phase scrambling		
	Mean	–CI	+CI	Mean	–CI	+CI	Mean	–CI	+CI
Human	3.43	3.22	3.61	6.02	5.81	6.16	58.44	53.43	60.40
VGG‐16	4.38	4.14	4.60	3.83	3.62	4.06	37.04	35.45	38.87
ResNet‐50	4.33	4.14	4.55	3.49	3.30	3.69	41.87	40.33	43.16
DenseNet‐201	4.14	3.89	4.37	3.73	3.59	3.91	43.12	41.97	44.57
AlexNet	5.12	4.78	5.44	3.45	3.17	3.74	33.70	31.66	35.77

Next, we proceeded to analyze the relative accuracy between CNN and human categorization performance, for each of the degradation levels, which were common to both humans and CNNs. For LSF pictures, significantly lower performance was observed for CNNs, specifically in all levels for AlexNet, in the three most degraded levels for VGG‐16 and ResNet, and in the two intermediate levels for DenseNet, corrected *p*s < .042, and for all levels for DenseNet *p* = .032. For HSF scenes, CNN categorization performance was significantly less accurate in all three least degraded levels, corrected *p*s < .001. For phase‐scrambled scenes, categorization accuracy of all CNNs was lower compared to humans in the three least degraded levels, corrected *p*s < .001.

**Fig. 3:**
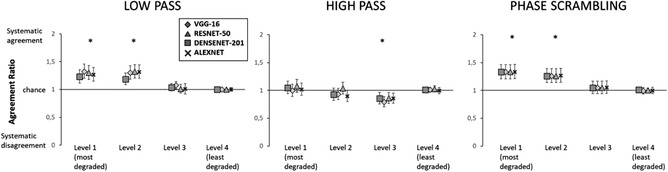
Agreement ratio in each of the experimental conditions, for all considered CNNs. * = significance at the .05 level. Error bars represent confidence intervals

Finally, we analyzed the agreement between humans and CNNs as indicated in the Method. In doing so, we computed the ratio between the observed and the expected agreement between humans and CNNs (Figure 3). For all CNNs, above chance agreement with humans was observed in the two most degraded levels of the low‐passed and phase scrambling conditions (corrected *p*s < .001), whereas significant disagreement was observed for all CNNs in the second least degraded level of the high‐passed condition (corrected *p*s < .05). No deviation from chance agreement was observed for the least degraded pictures; this apparently surprising result is a direct consequence of the definition of agreement that, as already discussed, tends to 1 in case of trivial tasks, that is, with ∼100% accuracy for both classifiers (a similar effect is seen in Tadros et al., 2019).

## 4 Discussion

The results of the first set of experiments indicated that, for all tested CNNs, accuracy was high for intact pictures but decreased rapidly with degradation. Similarly to humans, the decrease in accuracy followed a psychometric function, which, however, was shifted in the direction of less accurate categorization by CNNs than by humans. Additionally, humans and CNNs differed both in absolute accuracy and psychometric threshold, and systematic disagreement was observed in one HSF condition. Thus, humans and CNNs did not process visual information similarly, and more extreme differences between humans and CNNs were observed for high‐passed, compared with low‐passed or phase‐scrambled stimuli. These results suggest that the CNNs examined may show a functional HSF deficit, which ultimately affects their categorization performance and agreement with humans. Concerning phase‐scrambled scenes, the pattern of results followed that of low‐passed scenes, with a lower accuracy than humans on most levels, but a systematic agreement on the image‐level categorization in the two least degraded levels.

Importantly, the HSF deficit observed does not seem to depend on the depth of the tested CNN, as was observed with AlexNet (8 layers), VGG (16 layers), ResNet (50 layers), and DenseNet (201 layers), nor on the number of long‐range connections or convolutional structure, which also varied considerably among the examined CNNs. On the other hand, several alternative factors may contribute to the observed HSF deficit. First, CNNs may suppress fine‐grained information as pixel information is convoluted across the CNN's layers. If this is the case, then the observed HSF deficit should be a general characteristic of CNNs, and fine‐grained detail should not be expected to be sufficiently well processed in any condition. One consequence of this possibility would be that CNNs might aspire to be good models of human LSF processing, but less so for HSF processing, limiting their usefulness in the study of visual processing.

Alternatively, the observed HSF deficit may result from the statistical structure of visual stimuli. In real‐world scenes, the amplitude of LSFs is higher than the amplitude of HSFs by a function of 1/*f* (Burton & Moorhead, 1987; Field, 1987; Tolhurst, Tadmor, & Chao, 1992). In the image space, this amplitude–frequency relationship results in second‐order correlations between the intensities of pairs of pixels, increasing the redundancy in the visual input (Atick, 1992; Barlow, 1961, 2001; Shannon & Weaver, 1949; Field, 1987; Simoncelli & Olshausen, 2001). The visual system is adapted to these regularities, and it has been suggested that the organization of receptive fields at the earliest levels of the visual processing stream (retinal ganglion cells and lateral geniculate nucleus) serves the purpose of efficiently processing the visual input. One of the mechanisms suggested to increase coding efficiency is image decorrelation or whitening, implemented through the flattening of the spatial frequency spectrum in the retina and the lateral geniculate nucleus (Atick & Redlich, 1992; Croner & Kaplan, 1995; Graham, Chandler, & Field, 2006; Tan & Yao, 2009). Model and physiological data suggest that the sensitivity function of retinal ganglion cells increases with spatial frequency (Croner & Kaplan, 1995; Field & Brady, 1997; Graham et al., 2006). When a natural image, whose spatial frequency amplitude declines with a 1/frequency falloff, is convolved with an increasing sensitivity function, a flat spatial frequency amplitude is obtained; with a flat spatial frequency spectrum, second‐order correlations between pairs of points in the visual input are reduced (although not completely eliminated, as correlated firing still allows for plasticity phenomena to occur, e.g., in visual development; Graham et al., 2006; Wong, 2000).

In the next set of experiments, we investigated the possibility that image whitening, defined as the flattening of the spatial frequency spectrum, results in a more balanced use of low and high spatial frequency information in CNNs. More specifically, based on previous studies, we assume that the human visual system whitens natural pictures (such as the ones presented in the experiments described so far) at the outset, and, therefore, that further visual processing stages operate on a whitened visual input. If this reasoning also holds for CNNs, then preprocessing the visual input through whitening (Simoncelli & Olshausen, 2001) should determine a network performance that is more similar to that of human participants. Additionally, phase scrambling (which manipulates degradation without affecting spectral amplitude) may serve as a control for secondary effects of whitening on performance accuracy. If whitening determines an unspecific change in image categorizability, then its effects should not be restricted to (high and low) spatial frequency manipulation, but should also be evident on phase‐scrambled images.

## 5 CNN simulations with whitened pictures

### 5.1 Methods

**Fig. 4:**
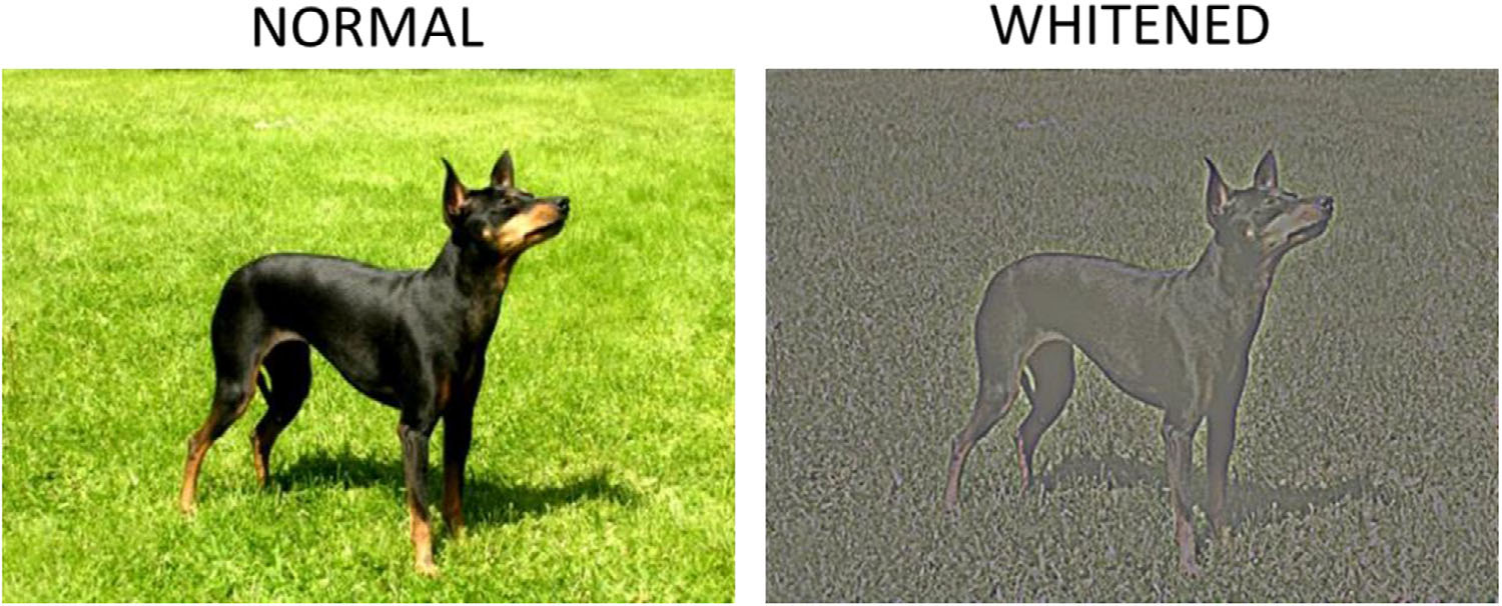
Example of an original (left) and whitened (right) picture

In the second set of experiments, all pictures were preprocessed in a whitening stage prior to CNN fine‐tuning and testing. This was achieved by multiplying the frequency amplitude spectrum by a function (increasing with spatial frequency up to the Nyquist frequency), which balanced the relative amplitude of low and high spatial frequency and flattened the spatial frequency spectrum of the image while avoiding high‐frequency noise (Atick, 1992). An example of an original and filtered picture is reported in Figure 4.

### 5.2 Results

**Fig. 5:**
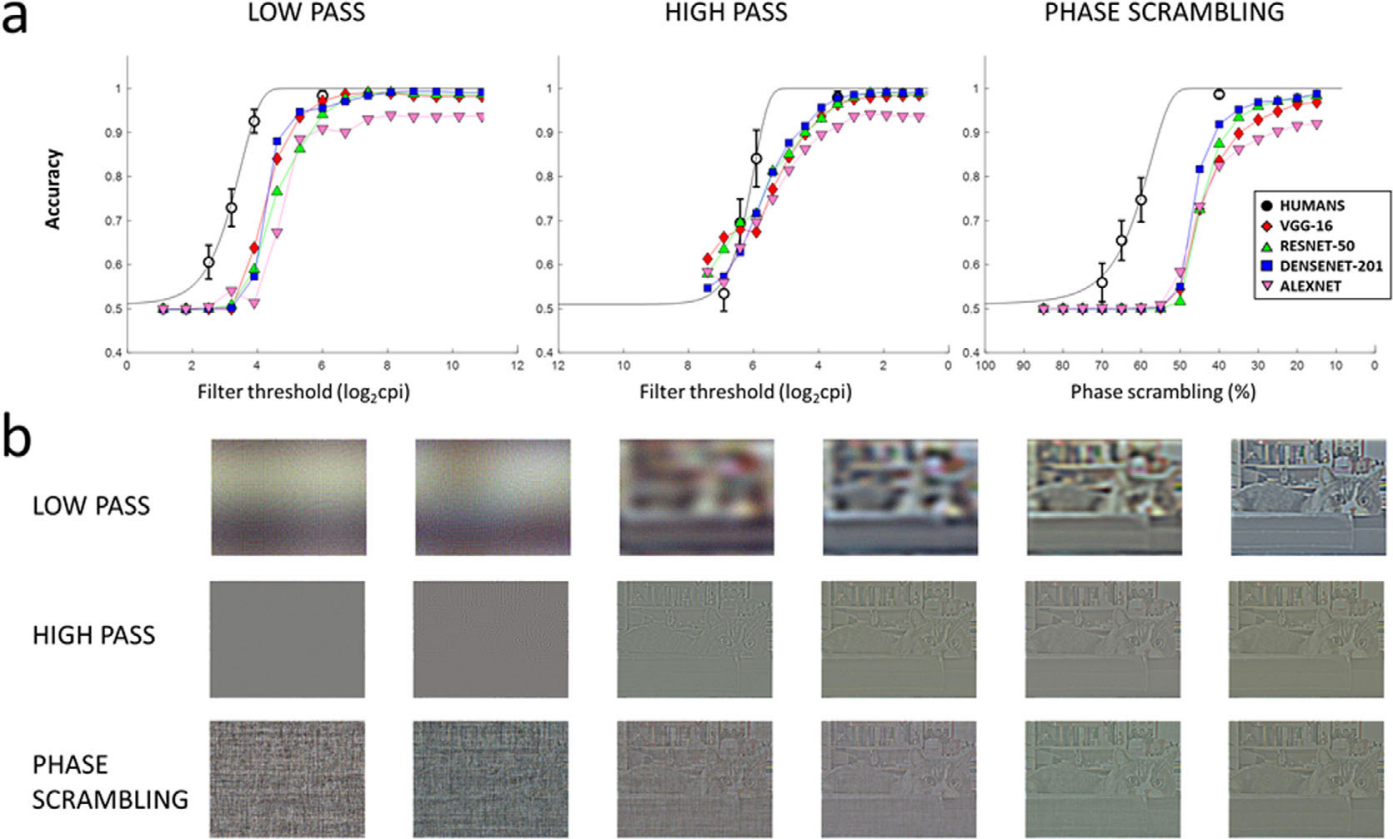
(a) Degradation‐accuracy functions for each of the considered CNNs and conditions. The straight black line represents the Weibull fit to the human data. Error bars for human data represent confidence intervals. (b) Examples of whitened pictures, for each degradation condition

**Fig. 6:**
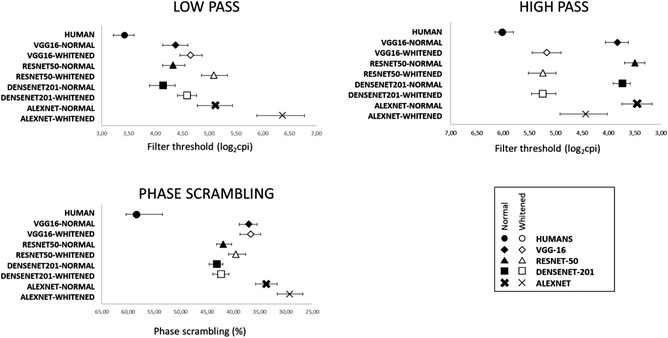
Psychometric threshold for all conditions, for normal pictures (filled symbols) and whitened stimuli (white symbols). Error bars represent confidence intervals

Results for whitened pictures are reported in Table 2 and Figure 5. The data can be summarized as follows: for HSF, adding a whitening stage determined accuracy functions that were closer to those of humans, and increased agreement. For LSF, whitening decreased categorization accuracy, albeit to a smaller extent compared with the HSF increase. For phase‐scrambled pictures, psychometric functions for whitened pictures were little or no different from functions for nonwhitened scenes. To facilitate the visual comparison between the experiments with normal and whitened pictures, Figure 6 visually summarizes the psychometric thresholds reported in Tables 1 (threshold for normal pictures, bold filled symbols) and 2 (thresholds for whitened pictures, light empty symbols).

**Table 2:** Threshold of the psychometric functions for whitened pictures

	Low pass			High pass			Phase scrambling		
	Mean	–CI	+CI	Mean	–CI	+CI	Mean	–CI	+CI
Human	3.43	3.22	3.61	6.02	5.81	6.16	58.44	53.43	60.40
VGG‐16	4.66	4.46	4.87	5.17	4.90	5.45	36.68	34.76	38.70
ResNet‐50	5.09	4.86	5.34	5.24	4.99	5.52	39.47	37.66	40.90
DenseNet‐201	4.59	4.41	4.77	5.25	5.00	5.46	42.34	40.79	43.88
AlexNet	6.37	5.89	6.78	4.43	4.02	4.92	29.28	26.80	31.61

Accuracy results followed those observed during the analysis of psychometric functions. Concerning low‐passed scenes, significantly lower accuracy for all CNNs compared with humans was observed in all levels, corrected *p*s < .016, except for the least degraded level in which VGG‐16 categorization did not differ from humans, corrected *p* = .704. With high‐passed pictures, lower accuracy of all CNNs compared with humans was restricted to the second least degraded level, corrected *p*s < .001, to the least degraded level for AlexNet, corrected *p* < .001, and exhibited a trend for DenseNet, corrected *p* = .057. On the other hand, significantly better performance of CNNs compared with humans was observed in the most degraded level for VGG‐16 and ResNet, corrected *p*s < .001. Concerning phase‐scrambled pictures, significantly lower accuracy was observed for all CNNs compared with humans in the three least degraded levels, corrected *p*s < .001.

To obtain a direct measure of the impact of whitening on each CNN, we carried out a further analysis comparing the categorization accuracy of each CNN when processing whitened versus nonwhitened pictures. For low‐passed pictures, a generally worse performance for whitened pictures compared with normal pictures was observed for all CNNs in the second least degraded level, corrected *p*s < .001, for the least degraded level for ResNet and the second most degraded level for DenseNet, *p*s < .001; additionally, a trend was observed in the same direction for the least degraded level and the DenseNet network, corrected *p* = .08. Concerning high‐passed scenes, whitening determined a better accuracy for all levels for DenseNet and VGG, for the three least degraded levels for ResNet, and for the two least degraded levels for AlexNet, all *p*s < .005. Finally, concerning phase‐scrambled pictures, whitening determined a worse performance for ResNet in the least degraded level, corrected *p* = .02.

As whitening determined opposite effects on the categorization of HSF and LSF pictures (Figures 5 and 6), we standardized psychometric thresholds in z‐points, to understand the difference between CNN and humans with normal and whitened pictures, and the change that was determined by whitening. For normal pictures, the overall difference compared to humans was 37.27 z‐points, which were reduced to 31.1 by picture whitening; this pattern was present for all CNNs except for AlexNet, which showed a modest effect in the opposite direction. This analysis is reported in Table 3, where for all CNNs a larger difference in comparison to humans is observed for HSF than for LSF original pictures. The pattern is reversed for whitened scenes, and the difference between the whitened and normal pictures is that whitening determined a closer to human performance of 13.17 z‐points for HSF scenes, a further away performance of 6.07 z‐points for LSF and 0.94 for phase‐scrambled scenes.

**Table 3:** Absolute difference (in z points) between threshold parameters for humans and CNNs, in all experimental conditions

				Phase	
		LPF	HPF	scrambling	Total
VGG‐16	Normal	6.97	20.73	7.47	35.17
	Whitened	11.39	6.19	7.96	25.54
	Whitened–Normal	–4.42	14.54	–0.48	9.64
ResNet‐50	Normal	6.65	23.89	5.79	36.33
	Whitened	11.53	7.33	6.97	25.83
	Whitened–Normal	–4.88	16.56	–1.18	10.5
DenseNet‐201	Normal	5.25	21.62	5.35	32.22
	Whitened	11.05	9.32	5.89	26.26
	Whitened–Normal	–5.80	12.30	–0.54	5.96
AlexNet	Normal	12.37	24.34	8.64	45.35
	Whitened	21.52	15.06	10.19	46.77
	Whitened–Normal	–9.16	9.27	–1.54	–1.43
Total	Normal	7.81	22.65	6.81	37.27
	Whitened	13.87	9.48	7.75	31.10
	Whitened–Normal	–6.07	13.17	–0.94	6.17

Note: For each condition, the Whitened–Normal subtraction is reported, which indicates the extent to which whitened thresholds are closer to humans (positive values) or further away (negative values), compared with thresholds for normal pictures.

**Fig. 7:**
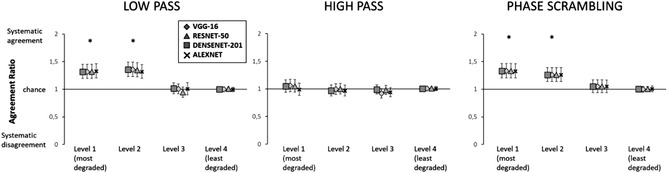
Agreement ratio in each of the experimental conditions, for all considered CNNs. * = significance lower than the .05 level. Error bars represent confidence intervals

Concerning image‐level agreement between humans and CNNs (Figure 7), the pattern of results for low‐passed and phase‐scrambled scenes was identical to that observed with nonwhitened images, and systematic agreement was observed in the two most degraded levels, corrected *p*s < .001. For high‐passed scenes, no systematic disagreement was observed in any condition, corrected *p*s > .26.

### 5.3 Discussion

The most important result of the second set of experiments is that the HSF deficit which was originally observed with normal pictures disappeared with whitened pictures. Performance for HSF scenes was close to that of human participants, while performance for LSF scenes showed a slight decrease compared to accuracy for nonwhitened pictures; this decrease was not as pronounced as the performance increase for HSF (Table 3). Moreover, analysis at the image level paralleled that at the group level: agreement between humans and CNNs with HSF scenes increased when the visual input was whitened.

## 6 General discussion

Here, we observed that the four CNNs tested approximated human two‐class categorization most accurately when they operated on whitened visual input. CNN categorization relied more on LSF, but only when the input followed the usual 1/*f* amplitude–frequency relationship (Field, 1987), and this was reflected both by performance accuracy and image‐level agreement with human decision. With whitened pictures, accuracy increased for HSF scenes and decreased for LSF scenes, and the overall difference compared to human performance was reduced; the effects of phase scrambling were altered very little, or not at all, by whitening.

CNNs, like other visual systems, need to adapt to the regularities of the visual world in which they operate. Here, the best match between CNN and human two‐class categorization was observed with whitened scenes. Whitening is a form of adaptation of visual systems to the statistical regularity in the 1/*f* amplitude–frequency relationship in the visual input (Field, 1987; Simoncelli & Olshausen, 2001). Consistently, a previous study (Cichy et al., 2016) indicated that significant spatio‐temporal hierarchical relationships between a CNN and the visual brain were only observed when the CNN was trained using natural scenes, but not when it was trained with unecological scenes, with noise, or when it was untrained (i.e., initialized with random weights). Similarly, a previous study reported that unsupervised training of a deep convolutional network with whitened natural images resulted in internal receptive fields, which responded to letter symbols (Testolin, Stoianov, & Zorzi, 2017). This result was taken as suggestive that the choice of letter primitives that constitute human written languages may be a result of the adaptation to the visual world in which humans operate, and that this process can be replicated in a DNN framework. Moreover, another study indicated that convolutional networks which were trained using natural images developed sensitivity not only to physical (pixel‐wise) stimulus features, but also to perceptual features and higher‐order category memberships (Kubilius et al., 2016). When degraded stimuli were taken into account, previous studies observed that fine‐tuning or training CNNs with degraded, rather than intact, pictures, resulted in a better performance with pictures of the same degradation type (Dodge & Karam 2019; Geirhos et al., 2018; Vasiljevic, Chakrabarti, & Shakhnarovich, 2016; Zhou, Song, & Cheung, 2017). However, here, we observed a more general result, namely that a general preprocessing stage which was inspired by the primate and human visual processing resulted in a more balanced use of visual information, that is, a more efficient use of HSF, a small decrease in accuracy with LSFs, and almost no change for phase‐scrambled pictures.

### 6.1 Use of information in visual categorization

Concerning the use of information in the present task, here we examined human performance in a two‐class superordinate categorization task. While some studies have suggested that LSFs drive the analysis of the visual input (Sanocki, 1993), others have emphasized the role of high‐frequency elements (Berman et al., 2017).^1^ For instance, several studies that examined emotional reactions suggested that emotional responses are driven by low‐frequency information that matches prelearned (in some cases, innate) survival‐critical representations (Vuilleumier, Armony, Driver, & Dolan, 2003; but see De Cesarei & Codispoti, 2013 and Pessoa & Adolphs, 2010, for critical reviews). However, when not only the physical availability of high and low spatial frequency information but also the semantic understanding of visual scenes was considered, no difference was observed between HSF and LSF stimuli in terms of emotional modulation of electrocortical activity (De Cesarei & Codispoti, 2011). Similarly, Berman and colleagues (2017) adopted a decoding approach to brain responses to visual scenes, filtered using low or high‐pass filters and scaled using contrast‐enhancing techniques (z‐score contrast scaling, compared to whole‐range scaling as used here and in previous studies; e.g., Loftus & Harley, 2005); it was observed that decoding from HSF stimuli was more accurate than from LSF and complete stimuli (Berman et al., 2017). Consistently, the present data emphasize the importance not only of low‐frequency, but also of high‐frequency visual information for human categorization. Moreover, by using a gradient of filter cutoffs which varied from extremely degraded to extremely clear, we observed that the human use of visual information, expressed as the increase in accuracy as a function of filter cutoff, increased similarly for high‐ and low‐ passed frequency.

Concerning CNNs, we observed that when keeping the same task and pictures as for human participants, the use of spatial frequencies varied according to the type of preprocessing (whitened or normal) that was carried out. In the first set of studies in which original pictures were presented to CNNs, we observed that CNN performance, and agreement with humans, for HSF pictures was inferior to that for LSFs, leading us to ask whether poor HSF visual processing reflects intrinsic limitations in CNNs. In contrast, the results of the second set of studies indicate that good accuracy and agreement with humans for HSF pictures can be achieved if pictures are whitened, demonstrating that HSF processing is not bound by intrinsic limitation of the examined CNNs. All in all, whitening did not increase the categorization accuracy by CNNs for intact scenes, but reduced the imbalance between the use of low and high spatial frequencies for categorization; this result seems to suggest that whitening can increase the classification accuracy of CNNs when flexibility is required, for example, when the perceptual appearance of an object may be unpredictably degraded by one of several types of degradation. A previous study suggests a possible reason for the difficulty of CNNs with high‐passed pictures, and for the better performance when whitened rather than nonwhitened HSF pictures are presented. In a recent study (Yosinski, Clune, Nguyen, Fuchs, & Lipson, 2015), visual representations in the internal layers of CNNs were examined, and it was observed that when no regularization was applied, internal representations were dominated by HSF noise, which was neither realistic nor interpretable (Yosinski, Clune, Nguyen, Fuchs, & Lipson et al., 2015; Nguyen et al., 2015). So, while internal HSF activation is produced, it is not diagnostic to the task at hand. In the experiments with nonwhitened pictures examined here, we focus on the performance of CNNs, and observe that in most HSF conditions, accuracy is considerably lower than that of humans, and that the difference compared to humans is larger for HSF than for LSF. It could be that the difficulty that CNNs show in achieving a good performance depends on the degree of internal HSF noise (Yosinski et al., 2015). This internal HSF noise has been suggested to be a consequence of the 1/*f* amplitude–frequency relationship of the pictures used for fine‐tuning (Yosinski et al., 2015, S1). Thus, it is possible that when whitening attenuates the 1/*f* slope, the internal HSF noise is reduced, yielding better performance for high‐passed whitened, compared with nonwhitened, pictures.

The effects of whitening were specific to the processing of the spectral amplitude, and whitening did not affect categorization of phase‐scrambled scenes. If whitening had determined an overall change in difficulty, then we would also have expected a change in performance in all conditions following image whitening. This, however, was not observed, suggesting that the effects of whitening on accuracy were specifically consequent to its effects on spectral amplitude, and not to changes in overall difficulty.

Here, we observed that humans and CNNs rely on different sources of information for the two‐class categorization task which was examined here. Moreover, we observed that differences between humans and CNNs were not restricted to overall accuracy, but extended to image‐level (dis)agreement with humans. More specifically, systematic agreement was observed for the most degraded LSF and phase‐scrambled levels for both normal and whitened pictures, suggesting that CNN performance in these conditions may draw on similar information as for human participants. Compared with previous results (Rajalingham et al., 2018), the present results indicate that, for some types of visual information, namely LSFs, significant image‐level agreement between human and CNNs can be observed. However, systematic disagreement for an HSF condition was observed with normal pictures, and this disagreement was eliminated by whitening. Recent studies investigated the bottom‐up (sensory and perceptual) and higher‐order semantic factors that can be a potential source of differences in the visual processing of humans and CNNs. When the texture and shape of faces and objects were manipulated, CNNs were more sensitive to changes in texture than in shape, while humans showed the opposite pattern (Baker, Lu, Erlikhman, & Kellman, 2018; Xu et al., 2018). Moreover, a recent study (Groen et al., 2018) indicated that human behavioral categorization depended both on perceptual factors, which could be modeled in hidden CNN layers (Cichy et al., 2016; Yamins et al., 2014), but also on other information which was not available to CNNs (i.e., object function, e.g., the fact that a bike can be used for transportation).

### 6.2 CNN architecture

The present study examined the performance of four CNNs (AlexNet, VGG, ResNet, and DenseNet) in the two‐class categorization of natural scenes. The results of the first experiments, using normal (nonwhitened) pictures, seemed to suggest that the considered CNNs exhibited a bias against HSFs (HSF deficit), perhaps because of a loss of fine‐grained details in the convolutional process. However, the experiments done using the same task and whitened stimuli argue against this possibility, as performance for HSF scenes was even better than for LSF pictures, in terms of difference compared to human thresholds (Table 3). This similar behavior was observed for networks varying in the number of total layers (from the 8 layers of AlexNet to the 201 layers of DenseNet), and in the organization of internal connections, supporting the idea that these kinds of networks learn similar sets of features (Oquab et al., 2014, Yosinski, Clune, Bengio, & Lipson, 2014, Zeiler & Fergus, 2013). On the other hand, these networks have important architectural similarities (convolution, max pooling, and regularization), and possibly also share similar limitations. Moreover, the organization of connections in the human brain is much more complex than the CNNs which were used here; for instance, feedback and lateral connections are present in the human brain, but are not present in the CNN models used here. Recently, it has been shown that the addition of feedback and lateral connections in recurrent convolution neural networks (RCNNs) resulted in superior performance compared with feedforward CNNs, in terms of overall performance and performance when to‐be‐recognized objects were occluded (Spoerer, McClure, & Kriegeskorte, 2017). Therefore, the present approach may be extended in future studies in order to compare the use of categorization by humans and RCNNs provided with lateral and top‐down (feedback) connections.

### 6.3 Limitations and further directions

In addition to the bottom‐up sensory and perceptual input, other factors may also play a role in shaping the similarity between the use of visual information by humans and CNNs. For instance, it is debated whether top‐down factors (e.g., attentional or emotional) can modulate early stages of perceptual processing (e.g., Morrison & Schyns, 2001; Schyns, 1998). In a previous study that examined this issue (De Cesarei & Loftus, 2011), we observed that human participants similarly used spatial frequencies over time while carrying out different versions of a categorization task (decision, forced choice, or confidence rating). Similarly, Loftus and Harley (2005) observed similar visual magnitude transfer functions when manipulating cognitive variables, such as priming, perceptual interference, and hindsight bias. However, other studies observed an advantage for basic categorization with long exposure times, and an advantage for superordinate categorization with short exposure times (Mack & Palmeri, 2015), and other studies observed a different use of spatial frequency information in different categorization tasks (categorization of emotional expression vs. expressiveness decision; Schyns & Oliva, 1999). Therefore, another promising avenue of research may generalize the present results, which describe the two‐class categorization of objects in natural scenes, to other categorization contexts, for instance, concerning subordinate categorization, object labeling or naming, or face processing.

In addition to the theoretical modeling of human vision, being able to simulate the successes and errors of human vision would be fruitful in several applied domains. For instance, it may be used in the design of urban or interior environments, to make safety signs visible even under degraded conditions, such as in smoke or night vision. These conditions may be simulated, for example, by phase scrambling or reducing the contrast of a virtual simulation of the environment, and a CNN may try to estimate the accuracy with which a sign is seen. In this respect, the research presented here provides a framework with which to test not only the overall accuracy of a CNN, but also its capability to simulate both correct and incorrect human perceptual decisions under specific visual conditions.

### 6.4 Conclusion

In a categorization task, performance accuracy of CNNs and humans increased according to a similar psychometric function; however, significant differences were also observed concerning the threshold parameters and the agreement between humans and CNNs. These differences were generally reduced when natural scene statistics (1/*f* amplitude–frequency relationship; Field, 1987) were considered. Vision does not only depend on the processing of shapes and contours, but also on the detection of statistical regularities, for example, clutter (De Cesarei, Loftus, Mastria, & Codispoti, 2017; Felsen & Dan, 2005; Greene & Oliva, 2009; Groen, Silson, & Baker, 2017; Scholte, Ghebreab, Waldorp, Smeulders, & Lamme, 2009; Torralba & Oliva, 2003). For humans and CNNs, visual regularities of the environment determine the development of internal representations (e.g., from receptive fields to more complex representations), which are selective for visual elements found in the environment, and these learned representations might transfer to novel visual stimuli, such as abstract symbols and letters (Kubilius et al., 2016; Testolin et al., 2017). At both a theoretical and practical level, adapting to natural scene statistics by whitening the visual input seems critical if a CNN is to be used as a model of human visual processing, and not only of the processing of some types of visual information (e.g., LSFs).

## Author contributions

A. De Cesarei, M. Lippi, and G. Cristadoro designed the study. All CNN simulations were performed by M. Lippi. A. De Cesarei and S. Cavicchi designed and ran the human experiment, analyzed all data, and wrote the initial draft. All authors contributed to the final draft and approved the final version of the manuscript for submission.

## Note

1 In addition to the spatial properties examined here, human vision also exhibits clear spatiotemporal properties. In studies presenting competing global and local information, temporal and functional precedence is observed for low spatial frequencies compared to high spatial frequencies (Schyns & Oliva, 1994; De Cesarei & Loftus, 2011; Kimchi, 1992; Navon, 1977). A critical variable in these studies was exposure time, which favored low spatial frequencies at short exposures (e.g., 100 ms or less followed by backward masking), and high spatial frequencies at long exposures (e.g., 1000 ms). Here, however, stimuli did not contain competing information (i.e., HSF and LSF information portrayed the same scene), and were presented for an intermediate time (800 ms), therefore, global precedence effects were neither expected nor observed. Moreover, in humans, eye and head movements bring the proximal projections of salient elements to the fovea. In CNNs, these spatial and temporal properties are often not modeled, and all visual information is presented at maximum resolution at the same time. In the future, other CNNs (e.g., recurrent convolutional neural networks) may help to improve current models by integrating known spatiotemporal properties of human vision.
